# Supplementary material for: The effect of successful educational actions in transition from primary to secondary school
Source: PLoS One. 2024 Jun 12;19(6):e0304683. doi: 10.1371/journal.pone.0304683 (PMC11168638; doi:10.1371/journal.pone.0304683)
Supplement: Attachment — Submitted filename: Response to Reviewers.pdf [file pone.0304683.s001.pdf]

Requirements or comments	Answers
Please ensure that your manuscript meets PLOS ONE's style requirements, including those for file naming. The PLOS ONE style templates can be found at https://journals.plos.org/plosone/s/file?id=wjVg/PLOSOne_formatting_sample_main_body.pdf and https://journals.plos.org/plosone/s/file?id=ba62/PLOSOne_formatting_sample_title_authors_affiliations.pdf	Done
Did you know that depositing data in a repository is associated with up to a 25% citation advantage (https://doi.org/10.1371/journal.pone.0230416)? If you've not already done so, consider depositing your raw data in a repository to ensure your work is read, appreciated, and cited by the largest possible audience. You'll also earn an Accessible Data icon on your published paper if you deposit your data in any participating repository (https://plos.org/open-science/open-data/#accessible-data).	As the project is nearing completion, we are curating the data to make it open access, after which it will be available on Zenodo.
Please state what role the funders took in the study.	Done. The clarification regarding the role of the funders was included in the manuscript and in the cover letter.
PLOS requires an ORCID iD for the corresponding author in Editorial Manager on papers submitted after December 6th, 2016. Please ensure that you have an ORCID iD and that it is validated in Editorial Manager.	Done
Please amend either the title on the online submission form (via Edit Submission) or the title in the manuscript so that they are identical.	Done
Please review your reference list to ensure that it is complete and correct. Any changes to the reference list should be mentioned in the rebuttal letter that accompanies your revised manuscript.	Done. The reference list is complete and correct. In particular, we have highlighted three references that were given extra space. We have also underlined two references that had the wrong year.
**Review Comments to the Author**	
Reviewer #1: I see most of the conversation are from the teacher, and only one of them is from a student. I believe it would be beneficial to include more input from the students.	Done. Quotes from five students were included in the findings section (pages 20, 21 and 26)
Reviewer #1. I believe the supplementary figures s1, s2 are not addressed. A more detailed discussion of Figures S1 and S2 would enhance the paper's comprehensiveness.	Done. A more detailed discussion of the figures was included on the Presentation of the case study section, page 18.
Reviewer #1. I believe including a comparison with schools not implementing SEAs would improve the robustness the argument.	In this respect, the aim of the study was not to compare the phenomena of other schools because that implies controlling all variables (type of study, participants -students, teachers -, time in which the study was conducted, context, etc.) but to analyse in a case study how the implementation of science-based practices that have proven to have a social impact contribute to the improvement of this particular phenomenon. Therefore, the quantitative data presented in the case study section have been taken into account and, in order to respond to the reasons for improvement, qualitative data have been collected based on the self-reporting of the main actors (teachers and students) of the school. Furthermore, the study is based on previous studies, mentioned in the literature review, which report on the social impact of Successful Educational Actions in various contexts.
The scope of this research is limited. It's confined within a single secondary school. This could be limiting the generalizability of the findings.	The research is intended as a case study and does not suggest a generalisation of the results obtained. At this point, the case study examines how the systematic implementation of these practices has impacted on the transition of students from primary to secondary

	school. The aim is to analyse and extrapolate key themes and findings that will help predict future patterns, illuminate pathways that can be applied to practice and provide a means to address the phenomena raised with greater clarity.
Reviewer #2: As in my view the paper can be accepted , the ethical approval and consent form participants are taken. However there needs to be some justification for the sample size and how were the participants selected .some latest software can be used for analysis of the Data.	Done. A justification for the sample size and how the participants were selected was included in the Data collection plan section (page 13).

Esther Roca Campos

Firmado digitalmente por Esther Roca Campos
Fecha: 2024.02.15 11:27:30 +01'00'
