# Supplementary material for: The effect of successful educational actions in transition from primary to secondary school
Source: PLoS One. 2024 Jun 12;19(6):e0304683. doi: 10.1371/journal.pone.0304683 (PMC11168638; doi:10.1371/journal.pone.0304683)
Supplement: Attachment — Submitted filename: Response to Reviewers.docx [file pone.0304683.s002.docx]

| **Requirements or comments** | **Answers** |
| --- | --- |
| **Review Comments to the Author** | |
| Reviewer #3:  1-Literature review – has to be upgraded and refined in reference of the innovation stated in the article. This can be achieved with additional literature review dated from the last two years. | Three studies published in the last two years have been added to the article (pages 3 and 10). |
| Reviewer #3:  2- Methods - It was specifically stated that the data collection was done through interviews, never-the-less, the article also includes quantitative data. In this case, the research is mix-method research, meaning it combines quantitative and qualitative methods. Therefore, proof and data have to be included in the methodology (method) chapter. | Although the article shows some quantitative data, this is not part of the fieldwork of the study. As explained in the section *Presentation of the case study*, the school was chosen for the case study because it has already shown positive results in terms of decreasing dropout rates, reduction of conflicts, etc. However, the study was traced through a qualitative case study, the fieldwork was conducted using qualitative techniques (interviews) and quantitative data were not analysed or collected for the purpose of the study, they were only taken into account for the selection of the case study. |
| Reviewer #3:  4- Analysis - In case of analysis, we look to test a change before and after an intervention. Therefore it is not enough to examine the change only according to frequencies, but it is necessary to use tests that include the value of significance. Meaning, without significance tests it is not possible to state any or a "significant" difference. | The study is not quantitative. The data presented correspond to official school data. At this point, the analysis is based on the perception of the students at the school regarding the contribution of the Successful Educational Actions in the transition process from primary to secondary school, in contrast to the official data mentioned above. |
| Reviewer #3:  5- Method - it is stated that the school has about a thousand students, however for this research, as stated in the article, only five students and ten teachers were selected for interviews. It is necessary to increase the number of students interviewed significantly, as well as the number of teachers. | The research is intended as a case study and does not suggest a generalisation of the results obtained. Although the school has more than a thousand students, the study focused on those with migrant or refugee background who have participated for at least one year in the proposed activities. At this point, rather than generalising the results through a display of quantitative data, the study demonstrates through the perspective of the beneficiaries how the systematic application of these practices has impacted on their transition from primary to secondary school. The results have therefore been analysed, which helps to illuminate pathways that can be pursued in practice and provide a means to more clearly address the challenges of school transition at this stage.  Therefore, the study focused on 1) students in the first or second year of secondary school because the research focuses on the transition from primary to secondary school, 2) the school decided to increase the implementation of these practices in these years because this is where the drop-out issue was highest, 3) as the study is qualitative, interviews were conducted using the saturation method, i.e. interviews were not conducted and analysed when they did not amplify information. |
| Reviewer #3:  6- It is advisable to add background variables such as ages. It is necessary to add the ages of the students who participated in the study, As the gaps might defer accordingly. Also, table 1 states the number of years of seniority of the teachers, however, no figure represents the data analysis. It is worth adding a reference to the contribution of this variable. | The data we have included are for the purpose of detailing the profiles of the study participants but are not intended to link these data to the results of the research. |
| Reviewer #3:  7- How were the respondents sampled in the study? It is necessary to add the information to the method chapter. | An explanation of how the participants of the study were selected is included in the Data collection plan section (page 13). |
| Reviewer #3:  8- The article should be proof read prior to publishing. There are several grammatical and language errors. | Correction of grammar and style for clarity done.  The corrected text provides more precise and clearer writing. |
| Reviewer #3:  9- Bibliography – Lack of uniformity in the bibliography. For example, at the beginning of the article the last names of the authors and the year of publication are written, and towards the end of the article the numbers of the articles as they appear in the final list appeared in parentheses. Also, there are unnecessary punctuation marks in some of the sources and in general there is a need for precision in the way of writing. | Done |
